# Inhibitory effect and mechanism of Rosiglitazone on M1 type polarization of central microglia in intracerebral hemorrhage mice based on JNK/STAT3 signaling pathway

**DOI:** 10.1002/brb3.3275

**Published:** 2023-10-14

**Authors:** Chenglei Chao, Yinghui Li, Quan Li, Guofeng Wu

**Affiliations:** ^1^ The Second Affiliated Hospital of Soochow University Suzhou Jiangsu Province P. R. China; ^2^ Department of Critical Care Medicine Changzhou Fourth People's Hospital Changzhou Jiangsu Province P. R. China; ^3^ Department of Emergency The Affiliated Hospital of Guizhou Medical University Guiyang Guizhou Province P. R. China; ^4^ Department of Emergency JinLing Hospital Medical School of Nanjing University Nanjing Jiangsu Province P. R. China

**Keywords:** intracerebral hemorrhage, JNK/STAT3, PPAR‐γ, Rosiglitazone

## Abstract

**Background:**

Intracerebral hemorrhage (ICH) seriously threatens the health of people. In addition, microglia M1 polarization was confirmed to be involved in the progression of ICH. Rosiglitazone was able to be used as an antidiabetic agent, which could activate PPAR‐γ, and PPAR‐γ was reported to inhibit inflammation in microglia. However, the detailed function of Rosiglitazone in ICH remains unclear.

**Methods:**

In vivo and in vitro experiments were used to test the function of Rosiglitazone in ICH. In addition, RT‐qPCR and western blot were performed to evaluate the mRNA and protein level of PPAR‐γ, respectively. Immunofluorescence staining was performed to detect the levels of CD206 and CD86, and ELISA was used to measure the levels of pro‐inflammatory cytokines.

**Results:**

PPAR‐γ was downregulated in ICH mice, whereas p‐JNK and p‐STAT3 were upregulated. Thrombin notably downregulated the level of PPAR‐γ in BV2 cells, whereas Rosiglitazone partially reversed this phenomenon. In addition, Rosiglitazone markedly reversed thrombin‐induced microglia M1 polarization. Consistently, thrombin‐induced inflammatory response in BV2 cells was abolished in the presence of Rosiglitazone. SP600125 (JNK/STAT3 inhibitor) greatly reversed thrombin‐induced M1 polarization in microglia, and GW9662 abolished the effect of SP600125. Meanwhile, Rosiglitazone could inactivate JNK/STAT3 pathway through the upregulation of PPAR‐γ. Furthermore, Rosiglitazone notably alleviated the symptom of ICH in vivo through inhibiting the apoptosis and mediating PPAR‐γ/JNK/STAT3 axis.

**Conclusion:**

Rosiglitazone could attenuate the inflammation in ICH through inhibiting microglia M1 polarization. Thus, our research would shed now lights on exploring new therapeutic strategies against ICH.

## Graphical abstract

Pathological signals of intracerebral hemorrhage. Created by Biorender.com.

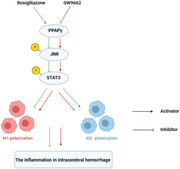

## 1 INTRODUCTION

Intracerebral hemorrhage (ICH) is mainly induced by hypertension and consists in the rupturing of blood vessels and leakage of blood into the brain parenchyma (Dicpinigaitis et al., 2022; Svedung Wettervik et al., 2022). Severe prognoses such as motor dysfunction, sensory paralysis, and impaired consciousness are observed in patients with ICH. Nowadays, the major strategies against ICH are drug administration and surgery, whereas the outcomes remain limited (Siedlecki et al., 2022). It was verified that the promotion of hematoma resolution and inhibition of hematoma expansion are considered the models of ICH; however, the translation to patients with ICH was not achieved yet (Wang et al., 2022; Wu et al., 2021). Therefore, it is essential to explore new therapeutic strategies for the treatment of ICH.

It has been confirmed that microglia could be polarized into M1 phenotype state (Jiang et al., 2022; Wang et al., 2023). In addition, M1 phenotype of microglia was considered pro‐inflammatory state, and inflammatory responses could induce the secretion of pro‐inflammatory cytokines (Kalkman & Feuerbach, 2016; Song et al., 2022). More importantly, M1 phenotype microglia could aggravate brain injury through the secretion of pro‐inflammatory cytokines (Gao et al., 2022; Ge et al., 2022). Activation of microglia was able to cause the progression of ICH (Deng et al., 2022; Wang et al., 2022). For instance, Duan et al. (2020) found that exosomal miR‐146a‐5p derived from bone marrow mesenchymal stem cells was able to attenuate the progression of ICH by alleviating M1 polarization of microglia; Li et al. (2021) suggested that Baihui (DU20)‐penetrating‐Qubin (GB7) acupuncture could regulate microglia polarization through the regulation of miR‐34a‐5p/Klf4 signaling in ICH rats. Thus, it is urgent to alleviate the M1 polarization of microglial cells for the treatment of ICH.

PPAR‐γ is a vital mediator in cell growth (Han et al., 2017; Janani & Ranjitha Kumari, 2015), and its upregulation could promote the cell proliferation (Li et al., 2018). In addition, the activation of PPAR‐γ was associated with the progression of inflammatory responses and cerebral infarction (Han et al., 2015). Meanwhile, Rosiglitazone (PPAR‐γ agonist) could drive the inflammatory responses during the progression of ICH, whereas it could also induce the adverse effect (including heart failure). For example, Krishna et al. (2021) found that Rosiglitazone could enhance myelination and neurological recovery in premature rabbits with ICH; Luo et al. (2021) suggested that Rosiglitazone could inactivate NF‐κB signaling in ICH. However, the mechanism underlying the function of Rosiglitazone in ICH remains further explored. On the other hand, JNK/STAT3 signaling is involved in the process of inflammation (Shi et al., 2017; Yoon et al., 2012), and its upregulation could promote the injury of organs. For instance, Sun et al. (2022) revealed that CTCF was able to activate JNK/STAT3 pathway to promote bronchial epithelial cell injury. Anti‐inflammatory effects of alcohol were related to the inactivation of JNK/STAT3 signaling (Mors et al., 2021). Meanwhile, a previous study suggested that PPAR‐γ in relation to JNK‐STAT signaling was able to prevent the progression of neurocomplications (Khera et al., 2022). However, the relation between PPAR‐γ and JNK/STAT3 signaling in ICH remains largely unknown. Besides, PPAR‐γ could reverse microglia M1 polarization, whereas JNK/STAT3 exhibited the opposite effect (Wan & Sun, 2019; Xu et al., 2022). Therefore, this research aimed to investigate the relation between Rosiglitazone and JNK/STAT3 pathway in microglia M1 polarization during the progression of ICH.

Based on the above backgrounds, it might be hypothesized that Rosiglitazone is able to attenuate the inflammation in ICH through suppressing microglia M1 polarization. Hence, we sought to investigate the mechanism by which Rosiglitazone regulates the inflammatory responses during the progression of ICH. We hope this work might suggest a new therapeutic target for ICH.

## 2 MATERIALS AND METHODS

### 2.1 Cell culture and treatment

Mouse microglia (BV2) was obtained from ATCC. Cells were maintained in DMEM (Thermo Fisher Scientific) containing FBS (10%, Thermo Fischer Scientific), penicillin, and streptomycin (1%) at 37°C, 5% CO_2_. To construct in vitro model of ICH, cells were exposed to thrombin as described previously (Hu et al., 2019). In addition, cells were divided into control, thrombin, thrombin + Rosiglitazone, thrombin + GW9662, thrombin + SP600125, and thrombin + GW9662 + SP600125 group. The experimental flowchart for in vitro experiments was listed in additional file (Figure 2).

### 2.2 Reagents

Rosiglitazone (cat. no. 122320‐73‐4), GW9662 (cat. no **M6191**), and SP600125 (cat. no S5567) were obtained from Sigma.

### 2.3 In vivo experiments

**FIGURE 1:**
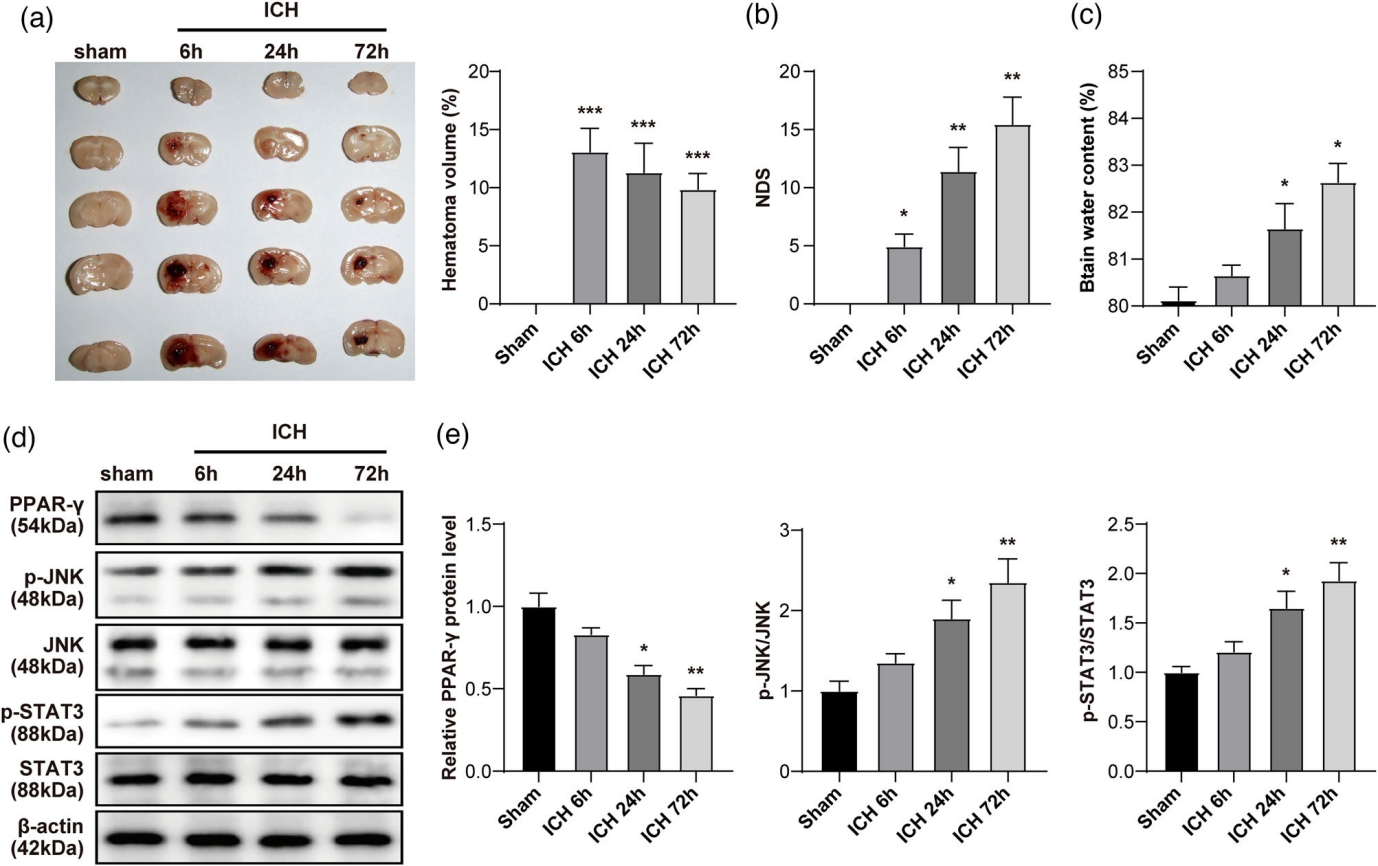
PPAR‐γ was downregulated in intracerebral hemorrhage (ICH), whereas JNK/STAT3 signaling was upregulated. (a) At the end of animal study, the brain tissues of mice were collected and pictured. The hematoma volume of tissues was detected. (b) The neurological deficit score (NDS) of mice was evaluated. (c) The brain water content of mice was calculated. (d) The protein levels of JNK, p‐JNK, STAT3, p‐STAT3, and PPAR‐γ in tissues of mice were assessed by western blot. (e) The relative expressions of p‐JNK and p‐STAT3 were quantified by normalizing to the total protein. The relative level of PPAR‐γ was quantified by normalizing to β‐actin. *n* = 5 per group. ^*^ *p* < .05, ^**^ *p* < .01 compared to sham.

To establish in vivo model of ICH, 20 C57BL/6 mice (*n* = 20, male, Vital River) were classified into four groups: sham (*n* = 5), ICH (6 h, *n* = 5), ICH (24 h, *n* = 5), and ICH (72 h, *n* = 5). In addition, to test the function of Rosiglitazone in ICH in vivo, 20 C57BL/6 mice (*n* = 40, male, Vital River) were used to establish in vivo model of ICH and classified into four groups: sham (*n* = 5), ICH (*n* = 5), ICH + Rosiglitazone (*n* = 5), and ICH + GW9662 (*n* = 5) group. In vivo model of ICH was constructed by autologous blood injection as recently reported (Luo et al., 2021). In brief, mice were anesthetized using 10% chloral hydrate (10 mg/kg) and fixed in a stereotaxic instrument (RWD). The scalp was sterilized using iodophors and 75% ethanol and incised along the skull midline. The surface of skull was cleaned by 3% H_2_O_2_, and then a hole was drilled at anterior–posterior 0.6 mm, lateral–medial 3 mm (from the Bregma). About 80 μL autologous blood was collected from the tail artery, and a total of 50 μL blood was injected into the cerebral caudate nucleus (−6.0 mm from the skull) using an automatic microinjection system (World Precision Instruments) at a rate of 10 μL/min. The needle syringe was left in place for 10 min before withdrawal. The skin was sutured and sterilized with iodophors. For mice in ICH + Rosiglitazone, mice were administrated orally with Rosiglitazone (3 days before the construction of ICH model, 3 mg/kg/day). Meanwhile, mice in ICH + GW9662 group were administrated with GW9662 (4 mg/kg/day) through the abdominal cavity after the establishment of ICH model. Then, they were sacrificed after 6, 24, or 72 h of ICH model establishment. The hematoma volume of brain tissues was detected. This research was approved by the ethics committee of The Affiliated Hospital of Guizhou Medical University (No. AHGMU20220604). The neurological deficit score (NDS) of mice was assessed in line with the recent report (He et al., 2020; Yu et al., 2020). The two‐part experimental flowchart of the in vivo experiments in this study was listed in additional file (Figure 1).

### 2.4 Brain water content investigation

After the whole brains of mice were harvested, the cerebral hemispheres were isolated immediately, and then the wet weights were evaluated. Subsequently, the cerebral hemispheres were dried. In detail, the brain tissues were dried in a 105°C oven to a constant weight, which was the dry weight. After 6, 12, or 72 h, the dry weight was recorded. The water content was calculated according to the following formula: (wet weight − dry weight)/wet weight × 100%. The protocol was in accordance with previously described (He et al., 2020).

### 2.5 Immunofluorescence

The brain tissues were extracted, and then slices were prepared as previously described (Im et al., 2019). The slices were incubated in PBS replenished 0.3% triton‐100 at ambient temperature for 10 min. Moreover, cells were fixed in paraformaldehyde (4%) for 20 min. Subsequently, the samples were blocked with 10% goat serum for 30 min and then incubated with anti‐Iba‐1 (Abcam, ab178846, 1:1000), anti‐CD86 (Abcam, ab239075, 1:1000), or anti‐CD206 (Abcam, ab64693, 1:1000) at 4°C overnight. After that, the samples were incubated with goat anti‐rabbit IgG (ab150077, 1:5000, Abcam) for 1 h at room temperature. Meanwhile, the nuclei were stained with DAPI (Beyotime) for 5 min. Finally, the images were captured under a light microscope (Olympus CX23; Olympus Corporation).

### 2.6 Western blotting

The protein was isolated from cells or tissues using RIPA (Beyotime). The protein was quantified with BCA kit (Beyotime) and then separated using SDS‐PAGE (10%, Beyotime). Subsequently, proteins were transferred onto PVDF membranes. The membranes were incubated with anti‐PPAR‐γ (ab178860, 1:1000), anti‐p‐JNK (ab307802, 1:1000), anti‐JNK (ab199380, 1:1000), anti‐p‐STAT3 (ab267373, 1:1000), anti‐STAT3 (ab68153, 1:1000), anti‐iNOS (ab178945, 1:1000), anti‐CD86 (ab239075, 1:1000), anti‐CD206 (ab64693, 1:1000), anti‐Arg1 (ab281603, 1:1000), anti‐Bax (ab32503, 1:1000), anti‐Bcl‐2 (ab182858, 1:1000), and anti‐β‐actin (ab8226, 1:1000) overnight at 4°C after blocking with skim, milk for 1 h. Then, the membranes were incubated with secondary antibody (HRP‐conjugated; ab288151, 1:5000) for 1 h. The protein bands were visualized by using ECL kit. These antibodies were provided with Abcam.

### 2.7 ELISA

The levels of IL‐1β (ab242234, 1:100), TNF‐α (ab183218, 1:100), IL‐10 (ab185986, 1:100), and TGF‐β (ab270739, 1:100) in BV2 cell supernatants or tissues were assessed by ELISA kit (Abcam) according to the instruction of manufacturer. In brief, the ELISA plates were pre‐coated with the primary antibodies overnight. After that, the supernatants were removed, and the plates were blocked with 10% FBS for 1 h. The plates were then treated with the samples for 2 h and then incubated with the secondary antibody (HRP‐conjugated, ab288151, 1:500) for 1 h. Then, plates were treated with HCL, and the absorbance (490 nm) was measured with a microreader. The procedure was in line with the previous reference (Xu et al., 2023).

### 2.8 TUNEL staining

Briefly, paraffin sections were washed, permeabilized, and then incubated with 50 μL TUNEL reaction mixtures in a wet box for 60 min at 37°C in the dark. For signal conversion, slides were incubated with 50 μL of peroxidase for 30 min at 37°C, rinsed with PBS, and then incubated with 50 μL diaminobenzidine substrate solution for 10 min at 25°C. Finally, the expression of apoptotic cells was observed under an optical microscope.

### 2.9 Statistical analysis

Three independent experiments were performed in each group. The data were expressed using mean ± SD. Western blot, RT‐qPCR, ELISA, and immunofluorescence staining were performed three times in each group. Consistently, other experiments were repeated three times. One‐way analysis of variance was used to compare the differences between multiple groups. *p* < .05 was considered a significant change.

## 3 RESULTS

### 3.1 PPAR‐γ was downregulated in ICH, whereas JNK/STAT3 signaling was upregulated

To detect the role of PPAR‐γ and JNK/STAT3 in ICH, in vivo experiments were applied. As shown in Figure 1a,b, the NDS was much upregulated in ICH mice than those in control, and the hematoma volume was limitedly absorbed with the time extension. The progression of ICH was able to increase the brain water content of mice in a time‐dependent manner (Figure 1c). In addition, the levels of p‐JNK and p‐STAT3 were time‐dependently upregulated in ICH mice, whereas the expression of PPAR‐γ exhibited the opposite trend (Figure 1d,e). To sum up, PPAR‐γ was downregulated in ICH, whereas JNK/STAT3 signaling was upregulated.

### 3.2 Rosiglitazone inhibited microglia M1 polarization through the upregulation of PPAR‐γ

**FIGURE 2:**
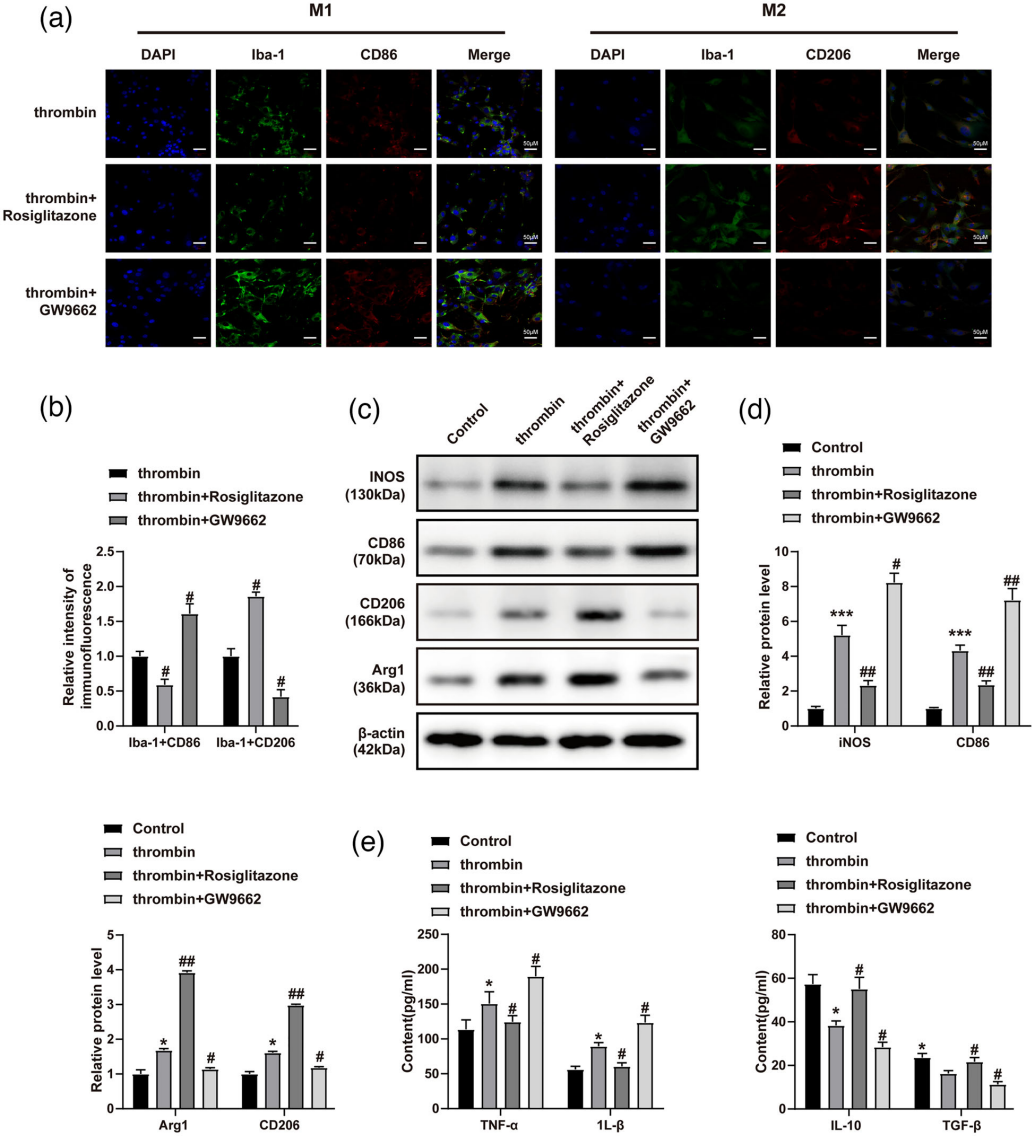
Rosiglitazone inhibited microglia M1 polarization through the upregulation of PPAR‐γ. BV2 cells were exposed to thrombin, thrombin + Rosiglitazone or thrombin + GW9662. (a and b) The levels of CD86 and CD206 in BV2 cells were assessed by immunofluorescence staining. The scale bar is 100 μm. (c) The protein levels of CD86, CD206, Arg1, and iNOS in BV2 cells were assessed by western blot. (d) The relative levels of CD86, CD206, Arg1, and iNOS were quantified by normalizing to β‐actin. (e) The levels of IL‐1β, TNF‐α, TGF‐β, and IL‐10 in BV2 cell supernatants were assessed by ELISA. ^*^ *p* < .05, ^**^ *p* < .01, ^***^ *p* < .001 compared to control. ^#^ *p* < .05, ^##^ *p* < .01 compared to thrombin.

It was known that Rosiglitazone was the activator of PPAR‐γ, whereas GW9662 exerted the opposite effect. Thus, to detect the effect of Rosiglitazone on M1 polarization in microglia, western blot was performed. As indicated in Figure 2a–d, thrombin significantly increased the levels of CD86, iNOS, CD206, and Arg1, and this phenomenon was further aggravated by GW9662. On the other hand, Rosiglitazone exerted the opposite effect. Furthermore, the levels of IL‐1β and TNF‐α in BV2 cells were notably increased by thrombin, whereas Rosiglitazone partially reversed this phenomenon (Figure 2e). In contrast, Rosiglitazone obviously rescued thrombin‐inactivated IL‐10 and TGF‐β in BV2 cells (Figure 2e). Taken together, Rosiglitazone inhibited the M1 polarization in microglia through the upregulation of PPAR‐γ.

### 3.3 Downregulation of PPAR‐γ reversed JNK/STAT3 signaling‐induced microglia M1 polarization

**FIGURE 3:**
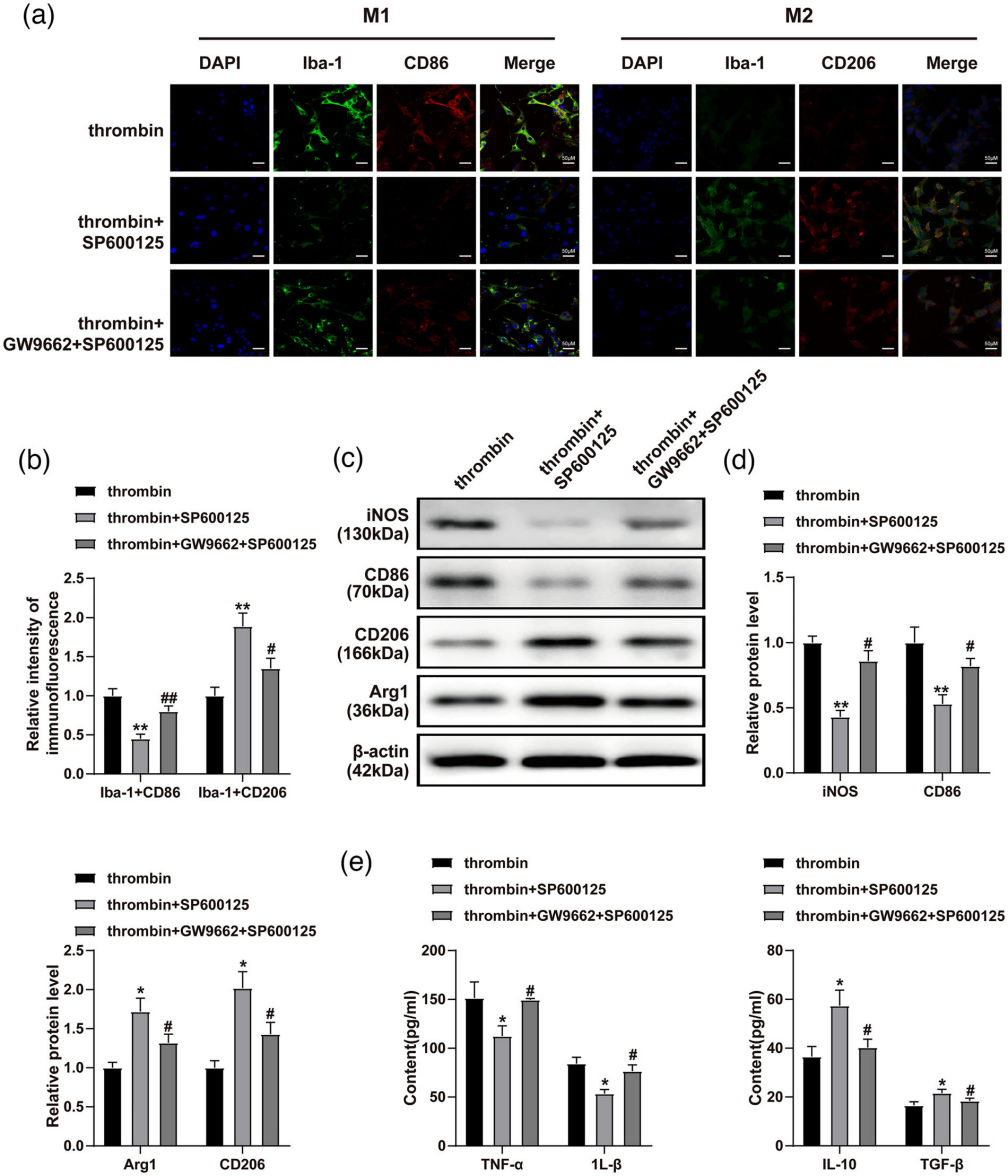
Downregulation of PPAR‐γ reversed JNK/STAT3 signaling‐induced microglia M1 polarization. BV2 cells were exposed to thrombin, thrombin + SP600125 or thrombin + SP600125 + GW9662. (a and b) The levels of CD86 and CD206 in BV2 cells were assessed by immunofluorescence staining. The scale bar is 100 μm. (c) The protein levels of CD86, CD206, Arg1, and iNOS in BV2 cells were assessed by western blot. (d) The relative levels of CD86, CD206, Arg1, and iNOS were quantified by normalizing to β‐actin. (e) The levels of IL‐1β, TNF‐α, TGF‐β, and IL‐10 in BV2 cell supernatants were assessed by ELISA. ^*^ *p* < .05, ^**^ *p* < .01 compared to thrombin. ^#^ *p* < .05 compared to thrombin + SP600125.

In order to explore the association between PPAR‐γ and JNK/STAT3 signaling in ICH, immunofluorescence staining was used. The data indicated that the inactivation of JNK/STAT3 pathway (SP600125) was able to inhibit the level of M1 phenotype markers (CD86, iNOS) and upregulate the level of M2 phenotype markers (CD206 and Arg1) in thrombin‐treated BV2 cells, whereas this phenomenon was restored by GW9662 (Figure 3a–d). In addition, the levels of IL‐1β and TNF‐α in BV2 cells were notably inhibited by SP600125; in contrast, SP600125 showed promotive effect on TGF‐β and IL‐10 (Figure 3e). GW9662 significantly reversed the inhibitory effect of SP600125 on microglia M1 polarization (Figure 3e). In summary, the downregulation of PPAR‐γ reversed JNK/STAT3–induced microglia M1 polarization.

### 3.4 Rosiglitazone inactivated JNK/STAT3 signaling through the upregulation of PPAR‐γ

**FIGURE 4:**
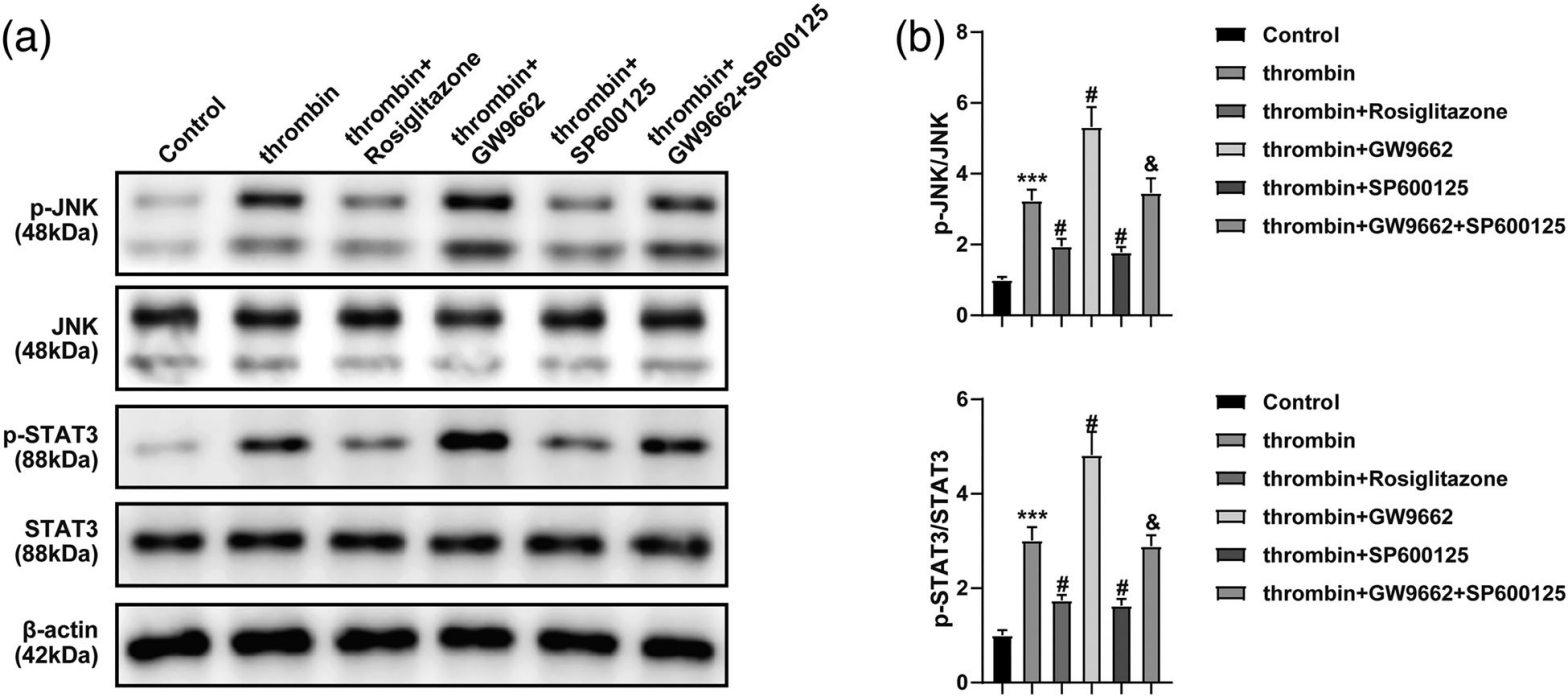
Rosiglitazone inactivated JNK/STAT3 signaling through the upregulation of PPAR‐γ. BV2 cells were exposed to thrombin, thrombin + Rosiglitazone, thrombin + GW9662, thrombin + SP600125, or thrombin + SP600125 + GW9662. (a) The protein levels of JNK, p‐JNK, STAT3, and p‐STAT3 in BV2 cells were assessed by western blot. (b) The relative expressions of p‐JNK and p‐STAT3 were quantified by normalizing to the total protein. ^***^ *p* < .001 compared to control. ^#^ *p* < .05 compared to thrombin. ^&^ *p* < .05 compared to thrombin + SP600125.

To further investigate the mechanism by which Rosiglitazone regulates JNK/STAT3 signaling, western blot was performed. As revealed in Figure 4a,b, thrombin‐caused upregulation of p‐STAT3 and p‐JNK in BV2 cells was greatly reversed by Rosiglitazone or SP600125 but aggravated in the presence of GW9662. Meanwhile, SP600125‐mediated p‐STAT3 and p‐JNK in thrombin‐treated BV2 cells were rescued by GW9662 (Figure 4a,b). Thus, Rosiglitazone was able to inactivate JNK/STAT3 signaling via activating PPAR‐γ.

### 3.5 Rosiglitazone alleviated the progression of ICH in vivo through inhibiting apoptosis and modulating PPAR‐γ/JNK/STAT3 axis

**FIGURE 5:**
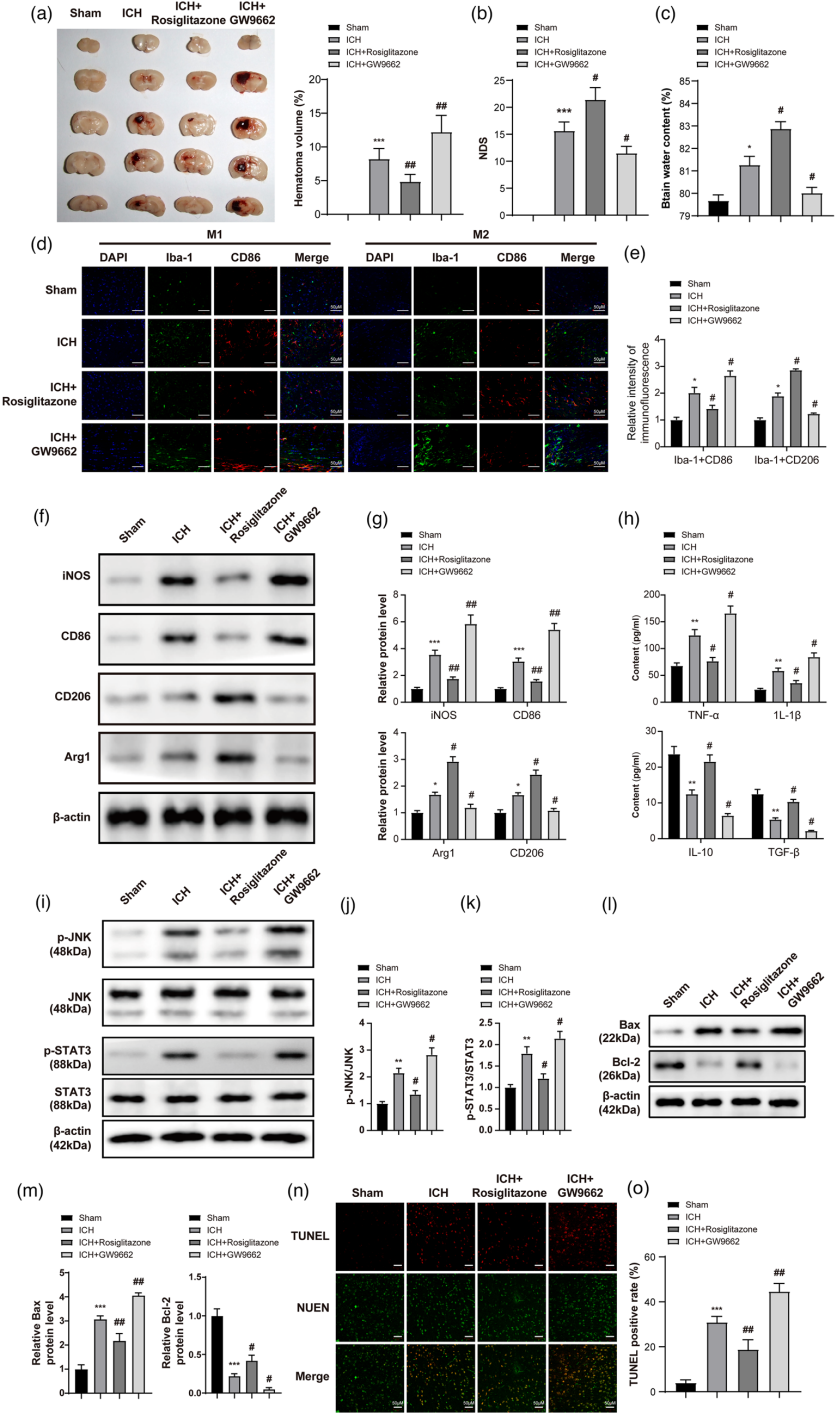
Rosiglitazone alleviated the progression of intracerebral hemorrhage (ICH) in vivo through mediation of PPAR‐γ/JNK/STAT3 axis. (a) At the end of animal study, the brain tissues of mice were collected and pictured. The hematoma volume of tissues was detected. (b) The neurological deficit score (NDS) of mice was evaluated. (c) The brain water content of mice was calculated. (d and e) The levels of CD86 and CD206 in mice were assessed by immunofluorescence staining. The scale bar is 100 μm. (f) The protein levels of CD86, CD206, Arg1, and iNOS in BV2 cells were assessed by western blot. (g) The relative levels of CD86, CD206, Arg1, and iNOS were quantified by normalizing to β‐actin. (h) The levels of IL‐1β, TNF‐α, TGF‐β, and IL‐10 in BV2 cell supernatants were assessed by ELISA. (i) The protein levels of JNK, p‐JNK, STAT3, p‐STAT3, and PPAR‐γ in mice were assessed by western blot. (j and k) The relative levels of p‐JNK and p‐STAT3 were quantified by normalizing to the total protein. (l and m) The protein levels of Bcl‐2 and Bax in mice were detected by western blotting. The relative levels of CD86, CD206, Arg1, and iNOS were quantified by normalizing to β‐actin. (n and o) The apoptosis in tissues of mice was tested by TUNEL staining. *n* = 5 per group. ^*^ *p* < .05, ^**^ *p* < .01, ^***^ *p* < .001 compared to sham. ^#^ *p* < .05, ^##^ *p* < .01 compared to ICH.

In order to further verify the function of Rosiglitazone in ICH, in vivo experiments were performed. As shown in Figure 5a–c, the hematoma volume, NDS, and water content in brain tissues of ICH mice were notably decreased by Rosiglitazone but further aggravated in the presence of GW9662. In addition, Rosiglitazone notably suppressed the level of M1 phenotype marker (CD86, iNOS) and increased the level of M2 phenotype marker (CD206 and Arg1) in ICH mice, whereas GW9662 exhibited the opposite effect (Figure 5d–g). The levels of IL‐1β and TNF‐α in ICH mice were notably inhibited by Rosiglitazone, whereas SP600125 upregulated the expressions of TGF‐β and IL‐10 (Figure 5h). Furthermore, the level of p‐STAT3 and p‐JNK in mice was greatly inhibited by Rosiglitazone SP600125 but upregulated by GW9662 (Figure 5i–k).

To explore the effect of Rosiglitazone on apoptosis during the progression of ICH, the apoptotic proteins were tested. The data suggested that ICH operation significantly upregulated the level of Bax and inhibited the expression of Bcl‐2, and this phenomenon was further aggravated by GW9662 (Figure 5l,m). In contrast, the effect of ICH operation was partially reversed by Rosiglitazone (Figure 5l,m). Meanwhile, ICH operation significantly induced apoptosis in brain tissues of mice, whereas this phenomenon was rescued by Rosiglitazone (Figure 5n,O). However, the apoptotic effect of ICH operation was greatly inhibited by GW9662 (Figure 5n,o). Taken together, Rosiglitazone alleviated the progression of ICH in vivo through inhibiting the apoptosis and modulating PPAR‐γ/JNK/STAT3 axis.

## 4 DISCUSSION

PPAR‐γ could play a vital role in inflammatory responses. For example, Li et al. found that PPAR‐γ was able to attenuate the inflammatory response in TNF‐α‐induced fibroblast‐like synoviocytes through interacting with *p*53 in rheumatoid arthritis (Han et al., 2022). PPAR‐γ could regulate the progression of ulcerative colitis through regulating TLR4/NF‐κB signaling (Wang et al., 2022). In this research, Rosiglitazone, a PPAR‐γ agomir, was found to inhibit the progression of ICH, and it was able to suppress the activation of JNK/STAT3 signaling. The previous studies found PPAR‐γ could positively regulate STAT3 and JNK signaling (Khera et al., 2022; Liu et al., 2023). Consistently, this study found that Rosiglitazone could mediate JNK/STAT3 signaling in ICH in vivo. Hence, our study first explored the effect of Rosiglitazone on JNK/STAT3 signaling in ICH, further suggesting the mechanism underlying the function of Rosiglitazone in ICH.

It was verified that microglia M1 polarization could induce the progression of ICH. For instance, Xu et al. (2022) suggested that Adiponectin alleviated the brain injury in ICH through inhibiting microglia M1 polarization; Hu et al. (2022) suggested that novel Nrf2 activator Omaveloxolone was able to modulate the M1 polarization of microglia and attenuate brain injury in ICH mice. Meanwhile, PPAR‐γ inhibited the M1 polarization of microglia (Zhou et al., 2022). Hence, PPAR‐γ was able to inhibit the progression of ICH through the inhibition of M1 polarization.

Microglia was the vital components of the immune system, which was the first line of defense against neuronal injury (Song et al., 2022). The activation of BV2 was confirmed to exist in M1 and M2 phenotypes. Meanwhile, M1 microglia was able to aggravate tissue injury through the secretion of pro‐inflammatory factors (IL‐1β, TNF‐α, etc.). The inhibition of M1 polarization in microglia was able to repair the tissue injury via declining the production of pro‐inflammatory factors (Cheng et al., 2021; Liu et al., 2021). Thus, the inhibition of microglia M1 polarization could inhibit the progression of ICH. Our study revealed that Rosiglitazone could reverse OGD/R‐induced M1 polarization in microglia, suggesting that Rosiglitazone could abolish thrombin‐induced inflammatory responses in BV2 cells via the suppression of M1 polarization.

The activation of JNK/STAT3 signaling promoted the inflammation, which was confirmed to regulate the M1 polarization of BV2 cells (Sun et al., 2021; Wan & Sun, 2019). In addition, our finding found the upregulation of PPAR‐γ was able to inhibit this signaling, which confirmed Rosiglitazone could inhibit microglia M1 polarization through inactivating JNK/STAT3 pathway. Meanwhile, Li et al. (2021) found that PPAR‐γ could attenuate neuron injury by the downregulation of CX3CR1 and M1 polarization, and this finding was similar to our research. The data of previous study revealed that CXC3R1 was the key mediator in microglia M1 polarization (Lin et al., 2019). Therefore, the similar function between CXC3R1 and JNK/STAT3 might contribute to the similarity between our study and Li et al.

Besides, previous studies have reported the relation between PPAR‐γ and other signaling in microglia M1 polarization. For example, antagonizing PPAR‐γ could facilitate the transition between M1 and M2 polarization of microglia via mediation of LKB1/AMPK pathway (Ji et al., 2018). According to the previous literature, the detailed relation between JNK/STAT3 and LKB1/AMPK is needed to be further explored in future.

Indeed, there are several limitations in this study as follows: (1) Some other targets of PPAR‐γ in ICH remain unexplored; (2) the relation between PPAR‐γ and JNK/STAT3 in ICH remains further explored. Hence, the other targets in ICH (as well as the association between PPAR‐γ and JNK/STAT3 in ICH) will be further explored in coming future.

Generally speaking, the novelty of this research was listed as follows: (1) The relation between PPAR‐γ and JNK/STAT3 in the progression of ICH was first suggested. Thus, this research was of great significance.

In summary, Rosiglitazone alleviates the progression of ICH through inhibiting microglia M1 polarization. Thus, our research might provide a theoretical basis for discovering a new strategy against ICH.

## AUTHOR CONTRIBUTIONS

**Chenglei Chao**: Conceptualization; writing–original draft; methodology; formal analysis. **Yinghui Li**: Data curation; investigation. **Quan Li**: Data curation; investigation. **Guofeng Wu**: Project administration; writing—review and editing.

## CONFLICT OF INTEREST STATEMENT

These authors declared no conflicts of interest in this study.

### PEER REVIEW

The peer review history for this article is available at https://publons.com/publon/10.1002/brb3.3275.

## Data Availability

All data generated or analyzed during this study are included in this article. The datasets used and/or analyzed during the current study are available from the corresponding author on reasonable request.
